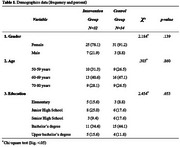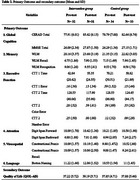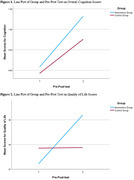# The Effect of Multi‐modal Cognitive Stimulation Program on Cognition and Quality of Life in Thai Older Adults with Mild Cognitive Impairment

**DOI:** 10.1002/alz70860_102050

**Published:** 2025-12-23

**Authors:** Phenphichcha Chuchuen, Phot Dhammapeera, Chavit Tunvirachaisakul

**Affiliations:** ^1^ Faculty of Psychology, Chulalongkorn University, Bangkok, Thailand; ^2^ Dementia Day Center, King Chulalongkorn Memorial Hospital, The Thai Red Cross Society, Bangkok, Thailand; ^3^ Department of Psychiatry, Faculty of Medicine, Chulalongkorn University, Bangkok, Thailand; ^4^ Center of Excellence in Cognitive Impairment and Dementia, Faculty of Medicine, Chulalongkorn University, Bangkok, Thailand

## Abstract

**Background:**

Mild cognitive impairment (MCI) is a growing global health issue, increasing dementia risk in older adults, including in Thailand. Multi‐modal Cognitive Stimulation Programs (MCSP), consisting of eight sessions, are effective non‐pharmacological interventions designed to slow cognitive decline and improve cognitive function and quality of life (QOL).

**Method:**

This study was a randomized controlled trial to evaluate the effects of an 8‐session MCSP on cognition and QOL in Thai older adults with MCI. Sixty‐six participants were divided into intervention (*n* = 32) and control (*n* = 34) groups. Data was collected using cognitive assessments (MMSE, CERAD, Color Trail Test, Digit Span) and QOL‐AD. Principal component analysis was used to congregate the scores from cognitive assessments. The analyses included chi‐square and t‐tests for demographic data and Generalized Estimating Equations (GEE) for cognition and QOL outcomes.

**Result:**

The study results below highlight the effects of MCSP on cognition and QOL. Demographic data showed no significant differences between the groups regarding age, gender, or educational background (see Table 1). No significant differences in cognitive scores and QOL were found between the intervention and control groups at baseline. After the MCSP intervention, the intervention group showed significantly higher scores than the control group (see Table 2). The study used GEE to analyze the impact of group, gender, age, education, and initial scores on score changes. These factors significantly predicted changes in scores, affecting overall cognition (see Figure 1). The result showed that Group has a significant effect on the cognitive score (*p* = 0.025), Age (*p* <0.001), Education years (*p* = 0.003), Initial scores (*p* <0.001), Gender and Group by Time show no significant effect. QOL scores measured by the QOL‐AD showed that Group (*p* = 0.007), Initial scores (*p* <0.001), and Group by Time (*p* = 0.011) had a significant effect (see Figure 2).

**Conclusion:**

This study shows that the CSP 8‐session intervention significantly improved cognition and quality of life in Thai older adults with MCI, as measured by cognitive assessments and QOL‐AD, compared to the control group.